# Impact of mean blood pressure and blood pressure variability after diagnosis of mild cognitive impairment and risk of dementia

**DOI:** 10.1111/jch.14391

**Published:** 2021-12-04

**Authors:** Adam de Havenon, Varsha Muddasani, Mohammad Anadani, Shyam Prabhakaran

**Affiliations:** ^1^ University of Utah Department of Neurology Salt Lake City Utah USA; ^2^ Department of Neurology Washington University St. Louis Missouri USA; ^3^ Department of Neurology University of Chicago St Louis Missouri USA

**Keywords:** blood pressure variability, dementia, hypertension, mild cognitive impairment, cognitive disorders/dementia, public health

## Abstract

Hypertension and increased blood pressure variability (BPV) are associated with the development of dementia. However, previous studies did not focus on the risk of dementia among participants with mild cognitive impairment (MCI) and controlled blood pressure level. To address this limitation, the authors performed a post‐hoc analysis of SPRINT MIND participants diagnosed with MCI (mean Montreal Cognitive Assessment score at diagnosis 16.1±3.1). The primary outcome was subsequent diagnosis of probable dementia. The exposure was mean blood pressure and BPV following MCI diagnosis until the end of follow‐up or a dementia event (mean follow‐up 2.6±1.2 years). The primary outcome occurred in 76/516 (14.7%) patients. The mean blood pressure was not significantly higher in participants who developed dementia. In the lowest quartile of BPV (systolic standard deviation), the rate of dementia was 8.5% (11/129), while in the highest quartile it was 21.7% (28/129). The highest quartile of systolic BPV had an adjusted hazard ratio for dementia of 2.73 (95% CI, 1.31–5.69) and for diastolic BPV it was 2.62 (95% CI, 1.26–5.47). In SPRINT MIND participants, the authors found that increased BPV after MCI diagnosis was associated with incident probable dementia during subsequent follow‐up.

## 1 INTRODUCTION

Hypertension is associated with a higher risk of dementia,^1^ as is increased visit‐to‐visit blood pressure variability (BPV).^2^, ^3^ Both hypertension and increased BPV lead to endothelial dysfunction and arterial stiffness over time,^4^ which is observed radiologically as an increase in white matter hyperintensities, microbleeds, and lacunae.^5^ Prior research has shown correlations between these radiological changes and the development of dementia.^6^ Because prior studies have not focused on the transition from mild cognitive impairment (MCI) to dementia, we performed an analysis of the Systolic Blood Pressure Trial ‐ Memory and Cognition in Decreased Hypertension (SPRINT MIND) cohort.

## 2 METHODS

We performed a post‐hoc analysis of the SPRINT‐MIND dataset.^17^ SPRINT‐MIND was an ancillary study to the parent SPRINT trial, which enrolled 9361 participants > 50 years old and with cardiovascular risk factors at 102 centers in the United States. SPRINT examined the benefit of a systolic blood pressure goal of < 120 versus < 140 on cardiovascular outcomes and, in the SPRINT MIND ancillary study, cognitive outcomes. Patients with dementia and a history of stroke were excluded from enrollment. We included participants enrolled in SPRINT MIND who had an outcome event of mild cognitive impairment (MCI) during the follow‐up period and at least four blood pressure measurements subsequent to the MCI event and prior to a dementia outcome or study completion.

IRB approval was not required for the deidentified dataset, which is publicly available.^7^ The primary outcome of our cohort study was incident probable dementia during the remainder of follow‐up after MCI diagnosis. The determination of probable dementia and MCI in SPRINT MIND relied on both screening and adjudication at follow‐up visits and planned cognitive testing at years 2 and 4, and study closeout when it was more than 1 year after the year four evaluation.^8^ In the parent SPRINT trial, seated blood pressures were recorded by trained study personnel recorded at study visits according to a protocol.^9^ The blood pressure used in the present study is a single value per visit that is the average of three seated blood pressures.

The study exposure was the mean blood pressure and BPV following MCI diagnosis until either the end of follow‐up or a dementia event. Because prior research has suggested that the adjudication of BPV improves with measurement using multiple methodologies, BPV was measured as standard deviation, coefficient of variation, residual standard deviation, average real variability, and successive variation. We fit a Cox proportional hazards model to the outcome of dementia. The Cox model was adjusted for age, sex, race, education, SPRINT randomization arm, history of cardiovascular disease, hypertension, diabetes, and mean blood pressure (except models fit to mean blood pressure). These covariates were selected based off prior research suggesting their possible confounding effects.^10^, ^11^ We verified the proportional hazards assumption of our Cox models by testing the Schoenfeld residuals.

## 3 RESULTS

Of the 640 patients who developed MCI in SPRINT MIND, we excluded 58 participants who had no further follow‐up and 66 who had less than four blood pressure measurements after MCI diagnosis. The remaining cohort of 516 participants had a mean±SD age of 73.8±8.7 years, was 66.2% male, 47.1% white, and mean Montreal Cognitive Assessment score at MCI diagnosis was 16.1±3.1 (Table 1). The mean±SD number of blood pressure measurements was 8.7±3.8 and mean±SD duration of follow‐up after MCI diagnosis was 2.6±1.2 years. The primary outcome of probable dementia was observed in 76/516 (14.7%). The mean systolic and diastolic blood pressure was not significantly higher in participants who developed dementia versus those who did not, but all measures of BPV were higher (Table 1). In the lowest quartile of BPV (systolic standard deviation), the rate of incident dementia was 8.5% (11/129), while in the highest quartile it was 21.7% (28/129).

**TABLE 1:** Baseline demographics and blood pressure metrics in 516 patients diagnosed with mild cognitive impairment (MCI)

**Variable**	**No dementia during follow‐up** (*n* = 440)	**Dementia during follow‐up** (*n* = 76)	***p* value**
Age in years	73.4±8.7	76.3±8.2	0.007
Male sex	297 (67.5%)	44 (57.9%)	0.102
Race/ethnicity			0.124
White	201 (45.7%)	42 (55.2%)	
Black	171 (38.8%)	22 (29.0%)	
Hispanic	55 (12.5%)	12 (15.8%)	
Other	13 (3.0%)	0	
History of diabetes	7 (1.6%)	4 (5.3%)	0.041
History of hypertension	400 (90.9%)	66 (86.8%)	0.268
History of atrial fibrillation (*n* = 514)	27 (6.2%)	7 (9.2%)	0.324
History of cardiovascular disease	95 (21.6%)	15 (19.7%)	0.716
History of stroke	2 (0.5%)	0	0.556
Smoking (*n* = 514)			0.372
Never	219 (49.9%)	43 (57.4%)	
Past	180 (41.0%)	28 (37.3%)	
Current	40 (9.1%)	4 (5.3%)	
Retired	344 (78.2%)	60 (79.0%)	0.881
Education			0.071
<College/other	322 (73.2%)	52 (68.4%)	
College	66 (15.0%)	8 (10.5%)	
Grad school	52 (11.8%)	16 (21.1%)	
Montreal cognitive assessment score at MCI diagnosis (*n* = 515)	16.1±2.8	15.7±3.9	0.322
Randomized to intensive blood pressure arm	205 (46.6%)	28 (36.8%)	0.115
Number of blood pressure readings after MCI diagnosis	8.6±3.8	9.2±3.8	0.209
Years of follow‐up after MCI diagnosis	2.7±1.2	2.3±1.0	0.012
SBP mean	130.3±11.4	131.7±9.9	0.288
SBP SD	11.4±5.1	12.9±5.4	0.027
SBP CV	8.8±3.8	9.7±4.0	0.039
SBP rSD	11.3±5.4	12.5±5.7	0.069
SBP ARV	12.9±6.3	14.4±6.6	0.051
SBP SV	15.2±7.2	17.6±7.9	0.010
DBP mean	68.4±9.8	68.4±10.5	0.979
DBP SD	6.4±2.7	7.2±3.0	0.018
DBP CV	9.5±3.8	10.7±4.4	0.011
DBP rSD	6.4±2.8	7.1±4.0	0.038
DBP ARV	7.3±3.3	8.3±3.9	0.022
DBP SV	8.6±4.7	10.0±4.7	0.003

*Abbreviations*: SBP, systolic blood pressure; DBP, diastolic blood pressure; SD, standard deviation; CV, coefficient of variation; rSD, residual standard deviation; ARV, average real variability; SV, successive variation.

In the Cox models fit to the outcome of dementia, we did not find an association for mean systolic or diastolic blood pressure. However, both systolic and diastolic BPV, as continuous variables and when comparing the highest to lowest quartile, were associated with dementia (Table 2). For example, the highest quartile of systolic standard deviation had an adjusted hazard ratio for dementia of 2.73 (95% CI, 1.31–5.69) and for diastolic standard deviation it was 2.62 (95% CI, 1.26–5.47). Similar effect sizes were seen for the other metrics of BPV in the adjusted Cox models (Table 2).

**TABLE 2:** Cox proportional hazards models showing hazard ratios for the development of probable dementia during follow‐up in 516 patients diagnosed with mild cognitive impairment (MCI)

**Blood pressure metric**	**Hazard ratio** *	**95% CI**	***p* value**
SBP mean (per 1 mm Hg shift)	1.00	0.97–1.03	0.956
SBP mean top quartile	1.26	0.52–3.05	0.608
DBP mean (per 1 mm Hg shift)	1.01	0.98–1.04	0.544
DBP mean top quartile	1.14	0.48–2.69	0.768
SBP SD (per 1 mm Hg shift)	1.05	1.01–1.10	0.029
SBP SD top quartile	2.73	1.31–5.69	0.007
DBP SD (per 1 mm Hg shift)	1.11	1.02–1.21	0.016
DBP SD top quartile	2.62	1.26–5.47	0.010
SBP CV(per 1 unit shift)	1.07	(1.01–1.13)	0.032
SBP CV top quartile	2.11	(1.06–4.17)	0.033
DBP CV (per 1 unit shift)	1.08	(1.02–1.14)	0.010
DBP CV top quartile	1.82	(0.90–3.64)	0.094
SBP rSD (per 1 mm Hg shift)	1.04	(1.00–1.09)	0.067
SBP rSD top quartile	2.12	(1.05–4.30)	0.037
DBP rSD (per 1 mm Hg shift)	1.10	(1.02–1.20)	0.018
DBP rSD top quartile	2.51	(1.24–5.11)	0.011
SBP ARV (per 1 mm Hg shift)	1.05	(1.01–1.08)	0.014
SBP ARV top quartile	2.70	(1.37–5.31)	0.004
DBP ARV (per 1 mm Hg shift)	1.09	(1.02–1.16)	0.009
DBP ARV top quartile	2.05	(1.03–4.10)	0.043
SBP SV (per 1 mm Hg shift)	1.04	(1.01–1.07)	0.020
SBP SV top quartile	2.48	(1.22–5.03)	0.012
DBP SV (per 1 mm Hg shift)	1.08	(1.02–1.14)	0.005
DBP SV top quartile	1.46	(1.23–4.91)	0.011

* Adjusted for participant age, sex, race, education, SPRINT randomization arm, history of cardiovascular disease, hypertension, diabetes, and mean blood pressure (except models fit to mean blood pressure). .

*Abbreviations*: SD, standard deviation; CV, coefficient of variation; rSD, residual standard deviation; ARV, average real variability; SV, successive variation.

## 4 DISCUSSION

In hypertensive SPRINT MIND participants, we found that increased BPV after MCI diagnosis was associated with incident probable dementia during subsequent follow‐up, independent of mean blood pressure. The effect of increased BPV on the risk of probable dementia was consistent for multiple different methodologic approaches to BPV measurement and for both systolic and diastolic BPV. Increased BPV has previously been associated with the development of dementia and MCI,^2^, ^12^ but the transition from MCI to dementia has not been fully explored. These hypothesis‐generating results warrant replication because the reduction of BPV is not a recognized treatment target to reduce the burden of dementia and specific classes of antihypertensive medications, such as calcium channel blockers, have been shown to reduce BPV.^13^

Our study has several important limitations. The most important is that this is a post hoc analysis that was not designed to answer the specific research question and, therefore, the results should be considered hypothesis‐generating only. We also did not have uniform blood pressure measurements because they were dependent on study visits, which may have biased the results, although the number of measurements was not different in patients who developed dementia versus not. The limited number of blood pressure measurements after MCI diagnosis also prevented sensitivity analyses where we could explore the impact of using more blood pressure measurements to improve the precision of BPV's measurement. We were not able to examine neuroimaging mediators of the reported associations, such as white matter hyperintensity or cerebral atrophy,^6^ because brain MRIs were only available in a minority of patients. Finally, we do not have data on the subtype of dementia (ie, vascular vs. Alzheimer's), which would have been of interest.

The implication of our findings is that BPV reduction may be particularly beneficial in patients who develop MCI, as a means of preventing the progression to dementia. The rate of incident dementia was high in this cohort of participants with MCI (14.7% over 2.6 years). However, this is consistent with prior studies of patients with MCI. For example, prior meta‐analyses have shown that patients with MCI have a rate of incident dementia that is three to five times higher than patients with normal cognition and in a prospective longitudinal cohort of 524 patients in the Mayo Clinic Study of Aging who were diagnosed with MCI, 16.1% had dementia by year two of follow‐up, consistent with our event rate.^14^, ^15^ A similar cohort may be of particular interest for future clinical trials of BPV reduction. Although BPV reduction has not been proven to reduce the risk of dementia, if an intervention could reduce BPV in the highest quartile to that of the lowest, the assumed reduction in dementia events is large enough that a trial with hundreds, not thousands, of participants would be feasible.

## DISCLOSURES

Dr. de Havenon has received investigator initiated clinical research funding from Regeneron and AMAG pharmaceuticals.

## AUTHOR CONTRIBUTIONS

Drs. de Havenon, Muddasani, Anadani and Prabhakaran conceived of the research, drafted the manuscript, and provided critical edits. Dr. de Havenon performed all statistical analysis. Adam de Havenon: conception or design of the work; the acquisition, analysis, or interpretation of data for the work; drafting the work or revising it critically for important intellectual content; final approval of the version to be published; agreement to be accountable for all aspects of the work in ensuring that questions related to the accuracy or integrity of any part of the work are appropriately investigated and resolved.

Varsha Muddasani: interpretation of data for the work; drafting the work or revising it critically for important intellectual content; final approval of the version to be published; agreement to be accountable for all aspects of the work in ensuring that questions related to the accuracy or integrity of any part of the work are appropriately investigated and resolved. Mohammad Anadani: interpretation of data for the work; drafting the work or revising it critically for important intellectual content; final approval of the version to be published; agreement to be accountable for all aspects of the work in ensuring that questions related to the accuracy or integrity of any part of the work are appropriately investigated and resolved. Shyam Prabhakaran: conception or design of the work; interpretation of data for the work; drafting the work or revising it critically for important intellectual content; final approval of the version to be published; agreement to be accountable for all aspects of the work in ensuring that questions related to the accuracy or integrity of any part of the work are appropriately investigated and resolved.
